# Investigating treatment dose error due to beam attenuation by a carbon fiber tabletop

**DOI:** 10.1120/jacmp.v7i3.2247

**Published:** 2006-08-24

**Authors:** W. Kenji Myint, Malgorzata Niedbala, David Wilkins, Lee H. Gerig

**Affiliations:** ^1^ Ottawa Hospital Regional Cancer Centre Ottawa Ontario Canada

**Keywords:** radiotherapy, couch attenuation, dose error

## Abstract

Carbon fiber is commonly used in radiation therapy for treatment tabletops and various immobilization and support devices, partially because it is generally perceived to be almost radiotransparent to high‐energy photons. To avoid exposure to normal tissue during modern radiation therapy, one must deliver the radiation from all gantry angles; hence, beams often transit the couch proximal to the patient. The effects of the beam attenuation by the support structure of the couch are often neglected in the planning process. In this study, we investigate the attenuation of 6‐MV and 18‐MV photon beams by a Medtec (Orange City, IA) carbon fiber couch. We have determined that neglecting the attenuation of oblique treatment fields by the carbon fiber couch can result in localized dose reduction from 4% to 16%, depending on energy, field size, and geometry. Further, we investigate the ability of a commercial treatment‐planning system (Theraplan Plus v3.8) to account for the attenuation by the treatment couch. Results show that incorporating the carbon fiber couch in the patient model reduces the dose error to less than 2%. The variation in dose reduction as a function of longitudinal couch position was also measured. In the triangular strut region of the couch, the attenuation varied ±0.5% following the periodic nature of the support structure. Based on these findings, we propose the routine incorporation of the treatment tabletop into patient treatment planning dose calculations.

PACS numbers: 87.53.Dq, 87.53.Mr

## I. INTRODUCTION

The growing use of carbon fiber materials in radiation therapy is largely due to their high mechanical strength, low specific density, and radiotranslucence.^(1)^ These characteristics make carbon fiber materials ideal for the patient support assembly. Prior to the use of these materials for treatment couches, the patient support structure consisted of a table mounted on steel rails or stabilized with a steel spine down its center. For various gantry angles, these high‐density rails have the potential to attenuate the beam by more than 40%, making it an obvious obstacle to avoid during patient setup. Consequently, geometric methodologies were proposed to ensure that beam–couch intersections were detected and dealt with appropriately.^(^ ^2^ ^–^ ^4^ ^)^

The benefits of carbon fiber over steel alloys make the new material a welcome change in radiotherapy practice, but because of the general assumption that attenuation by the couch is negligible, the strategies developed to avoid the couch are now often ignored. There is still little literature regarding the effects of carbon fiber patient support structures on clinical dose distribution. In 1991, de Mooy^(1)^ characterized the properties of carbon fiber and introduced applications of the material for use in radiation therapy. Meara and Langmack^(5)^ added to this characterization by investigating the transmission and buildup characteristics in 5‐MV, 6‐MV, and 8‐MV beams for thin panels of carbon fiber in combination with a variety of other plastics. The majority of subsequent publications have focused on attenuation, buildup, and increased skin dose caused by immobilization devices and table inserts. de Ost et al.^(6)^ observed 1% attenuation through commercial carbon inserts in Co‐60 and 6‐MV beams, but also noted that the surface dose increased from 18% to 77% of maximum dose, and 21% to 55% of maximum dose, respectively. Higgins et al.^(7)^ validated de Ost et al.'s results and concluded that there is minimal attenuation due to the inserts but added that the magnitude of the increase in surface dose was relatively larger for smaller beam sizes. Butson et al.^(8)^ quoted similar results for a 6‐MV photon beam. Carl and Vestergaard^(9)^ suggested that carbon fiber materials with thicknesses greater than 100 mg/cm^2^ should be avoided for 4‐MV beams to reduce skin dose in cases when the cumulative dose exceeds 54 Gy to 60 Gy. McCormack et al.^(10)^ extended previously published results by examining beam attenuation by a carbon fiber couch insert at various gantry angles. It was found that a 6‐MV photon beam was attenuated 2% at normal incidence and up to 9% at oblique angles. In 2003, Vieira et al.^(11)^ quantified the effect of carbon fiber treatment couch rails in combination with assorted immobilization devices on beam attenuation, reporting up to 15% attenuation of a 6‐MV photon beam during head and neck treatments.

In this study, the attenuation of photon beams traversing obliquely through the carbon fiber support rails of a clinical treatment couch were examined. Attenuation of beam fluence was measured in‐air at two locations along the treatment couch under various conditions for both 6‐MV and 18‐MV photon beams. Dose reduction measurements were also made in‐phantom. By including the carbon fiber couch in the CT image of the phantom, the effect of its attenuation on dose distribution was calculated with a commercial treatment‐planning system (Theraplan Plus v3.8, Nucletron, Ottawa, ON, Canada). The calculations were compared to measured data to investigate the ability of the planning system to properly model the couch attenuation

## II. MATERIALS AND METHODS

**Figure 1:**
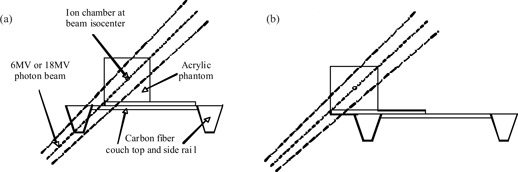
The experimental geometry for in‐phantom measurement of dose reduction, calculated by the ratio of readings (a) with and (b) without the couch. In‐air measurements were made under identical geometry except the phantom is replaced with brass buildup caps.

All measurements were performed on a Siemens Mevatron linear accelerator at The Ottawa Hospital Regional Cancer Centre. The treatment unit couch top was a Medtec indexed patient positioning system (IPPS^TM^) constructed with carbon fiber rails and grid panels. Measurements were made in‐air and in‐phantom using the geometry shown in Fig. 1. The in‐air measurements were made with an RK Chamber (model 8305) and Keithley Therapy Dosimeter (model 35040). For in‐air measurements, 0.4 cm and 0.8 cm thick brass caps provided buildup for 6‐MV and 18‐MV photon beam energies, respectively.^(12)^ The in‐phantom dose measurements were made at the center of a $20\times 20\times 20\;{\text{cm}}^{3}$ acrylic block phantom with a NE 2571 ion chamber and NE 2570 electrometer.

Attenuation was calculated by the ratio of the measurements taken under the two conditions shown in Fig. 1. The measurements were made with and without the carbon fiber couch rails attenuating the beam for $5\times 5\;{\text{cm}}^{2}$ and $10\times 10\;{\text{cm}}^{2}$ field sizes at both 6‐MV and 18‐MV photon energies delivering 100 monitor units. In all cases, the gantry angle was 225°, a beam orientation commonly used in standard radiation therapy and an angle that maximizes the thickness of carbon fiber material between the beam source and point of measurement.

**Figure 2:**
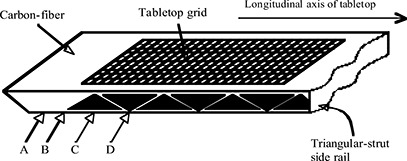
Longitudinal intersection of the beam with the treatment couch. At location A the entire field intersects the solid carbon fiber region of the couch. At locations B through D the beam transits the open strut region of the couch.

Figure 2 shows the longitudinal intersection points of the beam central axis with the couch. The in‐air measurements were taken at locations A and C, representing fields transiting through the solid and strut regions of the couch, respectively. The in‐phantom measurements were all taken at a location within the grid region between positions B and D. To investigate the longitudinal dependence of the beam attenuation, the experimental setup was shifted along the longitudinal axis of the treatment couch in increments of 1 cm beginning at location B and ending at location D.

The experiment described above was also modeled in Theraplan Plus version 3.8. A CT dataset (3 mm slice thickness with 3 mm spacing) of the entire phantom sitting on the Medtec therapy couch as shown in Fig. 1(a) was acquired on the Philips AcQsim large bore, single‐slice CT simulator (Philips Medical Systems, ON, Canada). The entire cross section of the treatment couch was included on each slice. The DICOM RT images were transferred to the treatment‐planning system, where dose calculations were performed using the standard Theraplan dose calculation model with inhomogeneity correction turned on. Calculations were performed for two cases: the first where the Med‐Tec couch was excluded from the calculation matrix and the second where it was included. In both cases, the CT tabletop was excluded from the treatment‐planning calculations.

## III. RESULTS

Attenuation of 6‐MV and 18‐MV photon beams by the carbon fiber side rails of the treatment tabletop was measured in‐air for both $5\times 5\;{\text{cm}}^{2}$ and $10\times 10\;{\text{cm}}^{2}$ fields. The results are shown in Table 1. As expected, the attenuation is field size‐ and energy‐dependent, the largest attenuation being 16.2% for smaller field sizes and lower energies. Attenuation is greatest near the head end of the tabletop (region A).

**Table 1:** In‐air and in‐phantom measurements of the effect of beam attenuation by the Medtec clinical treatment tabletop

		In‐air beam fluence attenuation (%)		In‐phantom dose reduction (%)
Energy	Field size	Solid region	Strut region	Strut region
6 MV	$5\times 5\;{\text{cm}}^{2}$	16.2	5.8	7.4
	$10\times 10\;{\text{cm}}^{2}$	15.3	5.5	6.8
18 MV	$5\times 5\;{\text{cm}}^{2}$	9.9	3.6	5.0
	$10\times 10\;{\text{cm}}^{2}$	9.3	3.5	4.7

Measurements were also made in‐phantom under the geometric conditions shown in Fig. 1(a) and (b). The effect of beam attenuation on the dose at depth in‐phantom shows field size and energy dependence similar to the in‐air observations. At the point of measurement, 100 cm source‐to‐axis distance, 14.1 cm depth, attenuation of the treatment field by the tabletop reduces the dose by up to 7.4% and is shown in the last column of Table 1.

**Figure 3:**
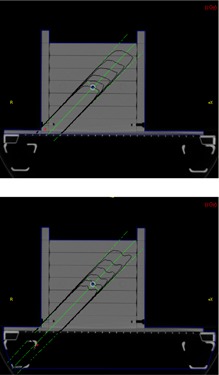
Theraplan isodose distributions calculated without (top) and with (bottom) the treatment couch included

To determine how well the planning system mitigates this error, comparison was made between the predictions of Theraplan Plus and measurements. The results are shown in Table 2. The first column under Dose Error compares measured and calculated dose when there is no couch in the beam, and represents how well our model in Theraplan Plus performs for this specific geometry. The second column compares the routine clinical situation where the table is physically present but ignored in planning. The final column shows the difference between measured and calculated dose when the tabletop is included in the Theraplan calculations. As shown, our model in Theraplan can only predict dose for the geometry in question to about 3%. The treatment table introduces a dose error up to 7.4% at the point of interest. When we introduce the tabletop into the calculations, Theraplan overcompensates for the table, but the total error becomes less than 1.4%. Figure 3 shows the Theraplan calculated dose distribution with and without the treatment couch.

**Table 2:** The difference between measured and calculated (TPP–Theraplan Plus) dose for 6‐MV and 18‐MV fields with and without attenuation from the treatment tabletop

Energy	Field size	Measured w/o table vs TPP w/o table	Dose error (%) Measured w/ table vs TPP w/o table	Measured w/ table vs TPP w/ table
	$5\times 5\;{\text{cm}}^{2}$	3.0	10.4	1.4
6 MV	$10\times 10\;{\text{cm}}^{2}$	2.3	9.1	1.3
	$5\times 5\;{\text{cm}}^{2}$	3.1	8.1	1.3
18 MV	$10\times 10\;{\text{cm}}^{2}$	2.3	7.0	0.4

## IV. DISCUSSION

Carbon fiber materials are often considered to be effectively transparent to high‐energy photons, particularly when compared to materials previously used for radiotherapy support structures, such as steel.^(5)^ This is reflected in the fact that no general purpose commercial treatment‐planning system provides a mechanism to account for the treatment couch during the planning process. It should be noted that Tomotherapy (TomoTherapy Incorporated, Madison, WI) planning systems do provide such a capability. For beams passing through thin carbon fiber meshes and table inserts, the assumption of transparency may be clinically acceptable with the exception of surface dose effects as described by Carl and Vestergaard^(9)^

Oblique treatment portals are commonly used (e.g., tangential breast, oblique lung, six‐field prostate, and conformal brain), and, depending on the setup geometry, beams may pass through the side rails of the treatment couch. Under these conditions, dose reductions in‐phantom of up to 7.4% were measured, while attenuation of up to 16.2% was measured for beams traversing the solid carbon fiber panel region at the superior end of the couch. If ignored, these are dose errors that could be clinically significant. For example, an oblique opposed pair for a lung boost could easily have the posterior beam passing through the couch rails. In this case, for equally weighted beams, the total localized dose error would be 3.7% or greater, depending on the location of the patient on the couch.

It has been shown that a small change in the dose can result in a much larger change in the local response of the tissue. For example, Sanchez‐Nieto and Nahum^(12)^ suggest that for prostate cancer a dose reduction of only 20% to 5% of the target volume can reduce the tumor control probability (TCP) by as much as 18%. Mijnheer et al.^(13)^ concluded that an overall accuracy of ±3.5% in the dose delivered to the ICRU reference point is required. Clearly, the localized dose error arising from ignoring couch attenuation exceeds the recommended dose uncertainty and may result in significant reductions in TCP. The assumption that carbon fiber is radiation transparent is not valid, and ignoring the attenuation can be clinically significant.

Mitigation of attenuation by beam avoidance is difficult, particularly with indexed couches and immobilization devices. One solution is to include the couch in the dose calculation, although this is not straightforward due to the structural differences between the CT couch and the LINAC couch. This study has shown that the Theraplan Plus planning system can predict the effect of the treatment couch on the dose distribution to better than 3%.

**Figure 4:**
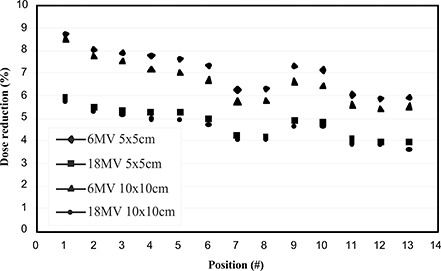
The dose reduction from attenuation by the tabletop at various positions along its length

We are currently exploring methods to routinely merge CT images of the treatment tabletop with patient CT scans. This involves replacing the portion of the image containing the CT couch with a previously acquired image of the treatment tabletop while maintaining the integrity of the patient data. The spatial relationship between the couch and the patient must be invariant between planning and treatment for this approach to be robust. This requirement is true for the lateral (IEC X) direction and to a lesser extent for the longitudinal (IEC Y) direction. Clearly, left‐to‐right shifts in patient position will result in the beam intersecting different components of the treatment couch with varying attenuations. Thus, the approach will require stringent patient indexing left to right. The variation in attenuation moving longitudinally along the couch has been explored. The results are shown in Fig. 4. It can be seen that the attenuation varies by ±1.5% along the entire region investigated, but by only ±0.5% over the portion of the couch defined by the tabletop grid. Thus, the requirement for indexing longitudinally is less stringent than left to right.

Radiotherapy techniques such as intensity‐modulated radiotherapy use smaller field sizes and a variety of gantry angles, increasing the need to address this clinical problem. Routine consideration of couch attenuation requires that patients be positioned reproducibly by methods such as indexing.

## V. CONCLUSION

This study investigated the dose error resulting from ignoring the beam attenuation by a carbon fiber treatment couch. It was found that neglecting this attenuation could result in clinically significant errors and that by including the treatment couch as part of the CT planning dataset, this error could be reduced significantly, even by a relatively simple dose calculation algorithm (pencil beam). We propose routine incorporation of the treatment couch as part of the treatment‐planning dataset and have determined that this would require patient immobilization and indexing of the immobilization device with the treatment tabletop.

## ACKNOWLEDGMENT

The Ottawa Regional Cancer Centre Foundation supported this research.
